# Supplementary material for: Effects of exercise intervention on falls and balance function in older adults: a systematic review and meta-analysis
Source: PeerJ. 2025 Oct 17;13:e20190. doi: 10.7717/peerj.20190 (PMC12536800; doi:10.7717/peerj.20190)
Supplement: Supplemental Information 1 [file peerj-13-20190-s001.docx]

**Retrieval strategy**

**PubMed**

#1 "Exercise" [Mesh]

#2 "Exercise Intervention"[Title/Abstract] OR "Physical Exercise"[Title/Abstract] OR "Physical Activity"[Title/Abstract] OR "Exercise"[Title/Abstract] OR "Yoga"[Title/Abstract] OR "Aerobic Exercise"[Title/Abstract] OR "Tai Chi"[Title/Abstract] OR "Resistance Training"[Title/Abstract] OR "Walking"[Title/Abstract] OR "Pilates"[Title/Abstract] OR "Qigong"[Title/Abstract] OR "Baduanjin"[Title/Abstract] OR "wu qin xi"[Title/Abstract] OR "liu zi jue"[Title/Abstract]

#3 "Aged"[Mesh]

#4 "aged"[Title/Abstract] OR "senior citizen"[Title/Abstract] OR "old people"[Title/Abstract] OR "geriatrics"[Title/Abstract] OR "elder"[Title/Abstract] OR "older adults"[Title/Abstract] OR "aged 60+"[Title/Abstract] OR "aging"[Title/Abstract] OR "senior"[Title/Abstract] OR "seniors"[Title/Abstract]

#5 "Accidental Falls" [Mesh]

#6 "accidental falls"[Title/Abstract] OR "falls"[Title/Abstract] OR "falling"[Title/Abstract] OR "accidental"[Title/Abstract] OR "accidental fall"[Title/Abstract] OR "fall"[Title/Abstract]

#7"Balance" [Mesh]

#8"balance"[Title/Abstract] OR "equilibrium"[Title/Abstract] OR "dynamic equilibrium"[Title/Abstract] OR "postural balance"[Title/Abstract] OR "dynamic balance"[Title/Abstract]

#9 randomized controlled trial [Publication Type]

#10 "randomized controlled trial"[Title/Abstract] OR "RCT"[Title/Abstract] OR "random"[Title/Abstract]

#9 #1 or #2

#10 #3 or #4

#11 #5 or #6

#12 #7 or #8

#13 #9 or #10

#14 #9 and #10 and #11 and #12 and #13

**Web of Science**

TS= “Exercise Intervention” or “Physical Exercise” or “Physical Activity” or “Exercise” or “Yoga” or “Aerobic Exercise” or “Tai Chi” or “Resistance Training” or “Walking” or “Pilates” or “Qigong” or “Baduanjin” or “wu qin xi” or “liu zi jue” AND TS=“aged” or “senior citizen” or “old people” or “geriatrics” or “elder” or “older adults” or “aged 60+” or “aging” or “senior” or “seniors” AND TS=“accidental falls” or “falls” or “falling” or “accidental” or “accidental fall” or “fall” AND TS="balance" or "equilibrium" or "dynamic equilibrium" or "postural balance" or "dynamic balance" AND TS=“randomized controlled trial” or “RCT” or “random”

**Embase**

('aged'/exp OR aged:ti,ab OR 'senior citizen':ti,ab OR 'old people':ti,ab OR geriatrics:ti,ab OR elder:ti,ab OR 'older adults':ti,ab OR 'aged 60+':ti,ab OR aging:ti,ab OR senior:ti,ab OR seniors:ti,ab) AND ('exercise'/exp OR 'exercise intervention':ti,ab OR 'physical exercise':ti,ab OR 'physical activity':ti,ab OR exercise:ti,ab OR yoga:ti,ab OR 'aerobic exercise':ti,ab OR 'tai chi':ti,ab OR 'resistance training':ti,ab OR walking:ti,ab OR pilates:ti,ab OR qigong:ti,ab OR baduanjin:ti,ab OR 'wu qin xi':ti,ab OR 'liu zi jue':ti,ab) AND ('falling'/exp OR 'accidental falls':ti,ab OR falls:ti,ab OR falling:ti,ab OR accidental:ti,ab OR 'accidental fall':ti,ab OR fall:ti,ab) AND ('balance'/exp OR 'equilibrium':ti,ab OR dynamic equilibrium:ti,ab OR postural balance:ti,ab OR dynamic balance:ti,ab:ti,ab OR balance:ti,ab) AND ('randomized controlled trial'/exp OR 'randomized controlled trial':ti,ab OR rct:ti,ab OR random:ti,ab)

**Cochrane Library**

#1 MeSH descriptor: [Exercise] explode all trees

#2 (“Exercise Intervention” or “Physical Exercise” or “Physical Activity” or “Exercise” or “Yoga” or “Aerobic Exercise” or “Tai Chi” or “Resistance Training” or “Walking” or “Pilates” or “Qigong” or “Baduanjin” or “wu qin xi” or “liu zi jue”):ti,ab,kw

#3 MeSH descriptor: [Aged] explode all trees

#4 (“aged” or “senior citizen” or “old people” or “geriatrics” or “elder” or “older adults” or “aged 60 and older” or “aging” or “senior” or “seniors”):ti,ab,kw

#5 MeSH descriptor: [Accidental Falls] explode all trees

#6 (“accidental falls” or “falls” or “falling” or “accidental” or “accidental fall” or “fall”):ti,ab,kw

#7 MeSH descriptor: [Balance] explode all trees

#8 (“balance” or “equilibrium” or “dynamic equilibrium” or “postural balance” or “dynamic balance”):ti,ab,kw

#9 MeSH descriptor: [Randomized Controlled Trial] explode all trees

#10 (“randomized controlled trial” or “RCT” or “random”):ti,ab,kw

#11 (#1 or #2) and (#3 or #4) and (#5 or #6) and (#7 or #8) and (#9 or #10)

**Summary of Findings and Quality of Evidence**

| Outcomes | Sample size | | Effect size [95% CI] | Risk of Bias | Inconsistency | Indirectness | Imprecision | Publication Bias | Quality of Evidence |
| --- | --- | --- | --- | --- | --- | --- | --- | --- | --- |
|  | Experimental group | Control group |  |  |  |  |  |  |  |
| MFES | 521 | 527 | 1.01, (0.63,1.40) | Serious (-1) | Serious (-2) | No | No | No | Low |
| NF | 477 | 473 | 0.32, (0.20, 0.51) | Serious (-1) | No | No | No | No | Medium |
| BBS | 665 | 633 | 0.92, (0.63,1.21) | Serious (-1) | Serious (-1) | No | No | No | Medium |
| TUGT | 956 | 929 | -0.62, (-0.79, -0.44) | Serious (-1) | Serious (-1) | No | No | No | Medium |
